# Assessment of muscle wasting in intensive care unit patients with and without COVID-19 using ultrasound imaging and bioimpedance analysis

**DOI:** 10.1186/s12871-026-03659-5

**Published:** 2026-02-03

**Authors:** Gintarė Šostakaitė, Erika Šalčiūtė-Šimėnė, Marija Svetikienė, Svetlana Danilenko, Andrius Klimašauskas, Jūratė Šipylaitė

**Affiliations:** 1 Clinic of Anaesthesiology and Intensive Care, Faculty of Medicine, Vilnius University, Vilnius, Lithuania; 2 Department of Mathematical Statistics, Vilnius Gediminas Technical University, Vilnius, Lithuania

**Keywords:** Intensive care unit acquired weakness, Muscle wasting, Muscle ultrasound, Bioelectrical impedance analysis, Phase angle, Handgrip dynamometry, COVID-19, Propensity score matching

## Abstract

**Background:**

Intensive care unit-acquired weakness (ICU-AW) is a common complication among critically ill patients, including those with COVID-19. While viral myopathy and established ICU-related risk factors predispose patients with COVID-19 to muscle dysfunction, few studies have directly compared muscle wasting and weakness between ICU populations with and without COVID-19 using both structural and functional assessment modalities.

**Methods:**

This was a small, non-concurrent, propensity score–matched ICU study which compared muscle wasting and strength in patients with and without COVID-19 who remained in the ICU for ≥ 7 days. Muscle thickness was assessed using ultrasound (US), body composition using bioelectrical impedance analysis (BIA), and functional strength using handgrip dynamometry. Measurements were performed on ICU days 1, 5, and 7. To reduce baseline differences, propensity score matching was applied using illness severity, nutritional risk, and mechanical ventilation parameters.

**Results:**

In total, 143 patients were included (101 without COVID-19, 42 with COVID-19). After propensity score matching, 23 pairs were analysed. US revealed significant within-group reductions in muscle thickness over time in both matched cohorts, with no statistically significant between-group differences. BIA-derived phase angle (PhA) values were consistently lower in patients with COVID-19; however, between-group differences in PhA change lost statistical significance after matching. Handgrip dynamometry revealed a significantly higher incidence of muscle weakness in patients with COVID-19 initially, but this difference was non-significant. Absolute and residual strength remained similar between groups.

**Conclusions:**

ICU patients both with and without COVID-19 experienced comparable degrees of muscle wasting and weakness when adjusted for baseline characteristics. ICU-AW appears more closely associated with the severity of critical illness and ICU treatments than with SARS-CoV-2 infection itself. US measurements appeared less affected by differences in fluid balance, whereas BIA-derived phase angle was more closely related to hydration status. Handgrip dynamometry provided a simple, objective measure of functional muscle strength at ICU discharge.

**Supplementary Information:**

The online version contains supplementary material available at 10.1186/s12871-026-03659-5.

## Introduction

Intensive care unit-acquired muscle weakness (ICU-AW) remains a persistent concern for patients during and after an intensive care unit (ICU) stay, with a prevalence of approximately 40% [1]. This condition is influenced by numerous factors, including patient-related characteristics (e.g., sex and pre-existing malnutrition), the cause and severity of critical illness (e.g., sepsis, systemic inflammatory response syndrome, multiorgan failure with a pronounced catabolic state, and stress-related insulin resistance), as well as ICU-related treatments (e.g., the use of sedatives, neuromuscular blocking agents, corticosteroids, prolonged immobilisation, and mechanical ventilation) [2–4]. 

Patients with severe COVID-19 requiring ICU admission often present with several established risk factors for developing ICU-AW [2]. In addition, there is growing evidence that the SARS-CoV-2 infection may be associated with skeletal muscle involvement and functional impairment [5–7]. Given the combination of viral myopathy and the high prevalence of ICU-AW risk factors, patients with COVID-19 may be at particularly elevated risk of developing severe and prolonged muscle dysfunction. However, muscle wasting and the associated muscle weakness have not been directly compared between critically ill populations with and without COVID-19 [8–11]. Assessing muscle wasting in critically ill patients is challenging because it requires monitoring changes over time while accounting for confounding factors such as the inflammatory response, fluid redistribution, and overall fluid balance—each of which can affect commonly used methods such as ultrasound (US) [12, 13] and bioelectrical impedance analysis (BIA) [14, 15]. Notably, BIA-derived phase angle (PhA) measurements are influenced not only by muscle loss but also by fluid status [16–18].

Several validated methods are available to assess ICU-AW in critically ill patients. The Medical Research Council Sum Score remains the most widely adopted clinical standard, with a total score of < 48 indicative of ICU-AW. However, given its limitations—including the need for full patient cooperation and the potential subjectivity of grading—simpler and more objective tools have gained popularity. Handgrip dynamometry, as demonstrated by Zhang et al. [19] and Ali et al., [20] has shown high sensitivity and specificity for diagnosing ICU-AW when using sex-specific cutoff values.

Given the limited understanding of muscle wasting patterns across different ICU populations, this study was performed to assess and compare muscle wasting, weakness, and residual strength in patients with and without COVID-19. A multi-modal assessment was performed, including US measurement of muscle thickness and BIA-derived PhA measurement for monitoring muscle mass, alongside digital handgrip dynamometry to evaluate functional muscle strength. To date, no direct comparison has been conducted between populations with and without COVID-19 using both structural (US and BIA) and functional (handgrip strength) muscle assessment tools. This study aims to address this gap.

## Materials and methods

### Study design and data collection

This small, non-concurrent, propensity score–matched ICU study was conducted in a mixed ICU at a tertiary teaching hospital in Lithuania (Vilnius University Hospital Santaros Clinics). The study included two distinct cohorts: patients without COVID-19 admitted to the ICU between 2018 and 2021, and patients with COVID-19 admitted to the ICU between 2021 and 2023). It represents a continuation of our prior research comparing BIA and US for the assessment of muscle mass loss in ICU patients [16]. Given the substantial baseline differences between groups, the primary analytical approach was a propensity score–matched comparison. Propensity score matching was therefore applied to create comparable cohorts of COVID-19 and non-COVID-19 patients, and the matched population constituted the primary analytical cohort for all inferential analyses.

The inclusion criteria for both groups were an age of ≥ 18 years, ICU length of stay (LOS) of ≥ 7 days, and Sequential Organ Failure Assessment (SOFA) score of ≥ 3. The exclusion criteria were an ICU LOS of < 96 h, the presence of a cardiac pacemaker, or limb amputation. Follow-up was limited to the first 7 ICU days, as this period typically encompasses the most pronounced catabolic response, fluid shifts and treatment intensity in critical illness. Our objective was to capture this early trajectory of muscle wasting, which is known to be most dynamic during the initial phase of ICU stay.

All patients were weighed using a bed scale, with adjusted body weight applied for those with obesity. Height was measured with a tape measure (with the patient lying flat) or estimated from the patient’s or their relative’s recall.

Data collected included demographic characteristics, comorbidities, admission diagnosis, Acute Physiology and Chronic Health Evaluation II (APACHE II) score, SOFA score on days 1 and 7 of the ICU stay, malnutrition risk score (Nutritional Risk Screening 2002 (NRS-2002]), use of sedatives and neuromuscular blocking agents, laboratory values (C-reactive protein, procalcitonin, albumin), fluid balance on days 5 and 7, and nutritional data (caloric and protein intake).

The primary outcomes were changes in muscle thickness as assessed by US, changes in PhA derived from BIA measurements, and muscle strength measured by dynamometry upon ICU discharge. The secondary outcomes were ICU LOS, duration of mechanical ventilation, ICU survival, and hospital survival.

This research was approved by the regional bioethics committee (Research No. 158200-17-954-459). All patients or their legally authorised representatives provided written informed consent.

During BIA and US measurements, the patients were positioned supine with arms abducted 15° from the trunk and legs spread at shoulder width, after resting flat for 10 min. Measurements were taken on day 1 (within 24 h of ICU admission), day 5, and day 7. All were performed by a single investigator.

Given the heterogeneity of critically ill populations, matching techniques such as propensity score matching (PSM) can help reduce baseline differences and enable more reliable comparisons of muscle-related outcomes.

### BIA

In BIA, the body’s impedance—comprising resistance (R) and reactance (Xc)—is measured to estimate body composition. When an alternating current passes through the body, cell membranes act as capacitors, creating a delay in current flow. This delay produces a phase difference between current and voltage, known as the PhA, which reflects cell membrane health and function. The PhA is considered indicative of nutritional status and malnutrition, and it has been shown to predict mortality, morbidity, and muscle function impairment, particularly reductions in muscle volume and strength.

The InBody S10 device (Biospace, Seoul, Korea) was used for assessment. This single-frequency, phase-sensitive BIA instrument applies a constant-frequency alternating current (400 µA, 50 kHz) and uses eight electrodes positioned on all four limbs, in accordance with the manufacturer’s recommendations (on both hands and between the anklebones and heels). The PhA (in degrees) was measured and recorded.

Differences in the PhA between days 1 and 5 and between days 1 and 7 were analysed, with day 1 measurements serving as the baseline.

### US

Muscle US is a non-invasive imaging technique that uses sound waves to visualise muscle structure and condition. It is widely employed in clinical practice to evaluate the effects of injury, inflammation, or other muscle-related conditions on muscle tissue.

For US assessment, the GE LOGIQ P9 machine (GE HealthCare Technologies Inc., USA) was used, equipped with a 10- to 12-MHz small-parts probe and a multifrequency linear-array probe in musculoskeletal mode. A water-soluble transmission gel was applied to the probe, and no compression was exerted during the procedure. Muscle thickness was measured with electronic callipers after obtaining a transverse image of the muscle. The biceps brachii was assessed with the palm facing upwards, focusing on a point two-thirds of the distance between the acromion and the cubital fossa. The rectus femoris and vastus intermedius were evaluated with the leg extended, at the midpoint between the anterior superior iliac spine and the patella. Three measurements were taken for each muscle, and the average was calculated to improve accuracy. Evaluation sites were marked with a skin marker to ensure consistency across subsequent measurements. Day 1 measurements of muscle thickness were used as the baseline, with values on days 5 and 7 expressed as percentages relative to the baseline.

Serial ultrasound and BIA measurements were planned on days 1, 5 and 7 of ICU stay. Patients who had fewer than three assessments were not included in the longitudinal change analyses. In total, 13 patients in the COVID-19 cohort and 37 patients in the non-COVID-19 cohort had incomplete datasets due to early ICU discharge, transfer or logistical constraints. Longitudinal analyses were therefore performed only in patients with complete paired measurements for the respective time points. No imputation of missing data was performed.

### Dynamometry

Muscle strength was assessed in survivors on the day of ICU discharge using a dynamometer (Jamar Plus Digital Hand Dynamometer; United Kingdom). Three measurements were taken with the dominant hand, with a 30-second pause between attempts. Patients were seated with the arm extended at the elbow. The average of the three measurements was recorded. Muscle weakness was defined as a force value of < 11 kg for men and < 7 kg for women. Residual muscle strength, expressed as a percentage from the norm for age and sex, was also calculated. Reference values were taken from the study by Landi et al., which reported dynamometer measurements in 11,448 participants [21]. 

### Statistical analysis

Quantitative variables were tested for normality using the Shapiro–Wilk test. If normality was confirmed, means were compared using parametric tests: Student’s t-test was used for independent samples, and the paired Student’s t-test was used for dependent samples. If normality was not met, non-parametric tests were applied: for independent samples, the Kruskal–Wallis test and the Mann–Whitney U test; for dependent samples, the Friedman test and the Wilcoxon signed-rank test.

For qualitative variables, associations between categorical variables were examined using the chi-square test. Differences were considered statistically significant at *p* < 0.05. Data analysis was carried out using IBM SPSS Statistics, version 26.

PSM was performed to reduce baseline differences between ICU patients with and without COVID-19. The propensity score was estimated using a logistic regression model including age, SOFA score on admission, NRS-2002 score, cumulative duration of mechanical ventilation over 7 days, and cumulative exposure to neuromuscular blockers. Body mass index was intentionally excluded from the model to improve overlap and matchability, given its disproportionate distribution between groups and its limited independent predictive value for ICU-AW.

Nearest-neighbour 1:1 matching without replacement was applied, using a calliper width of 0.3 standard deviations of the logit of the propensity score. This calliper was selected to optimise balance while maximising the number of matched pairs. In total, 23 matched pairs were obtained. All inferential analyses were conducted in the propensity-matched cohort, which was considered the primary analytical population of the study.

## Results

**Fig. 1:**
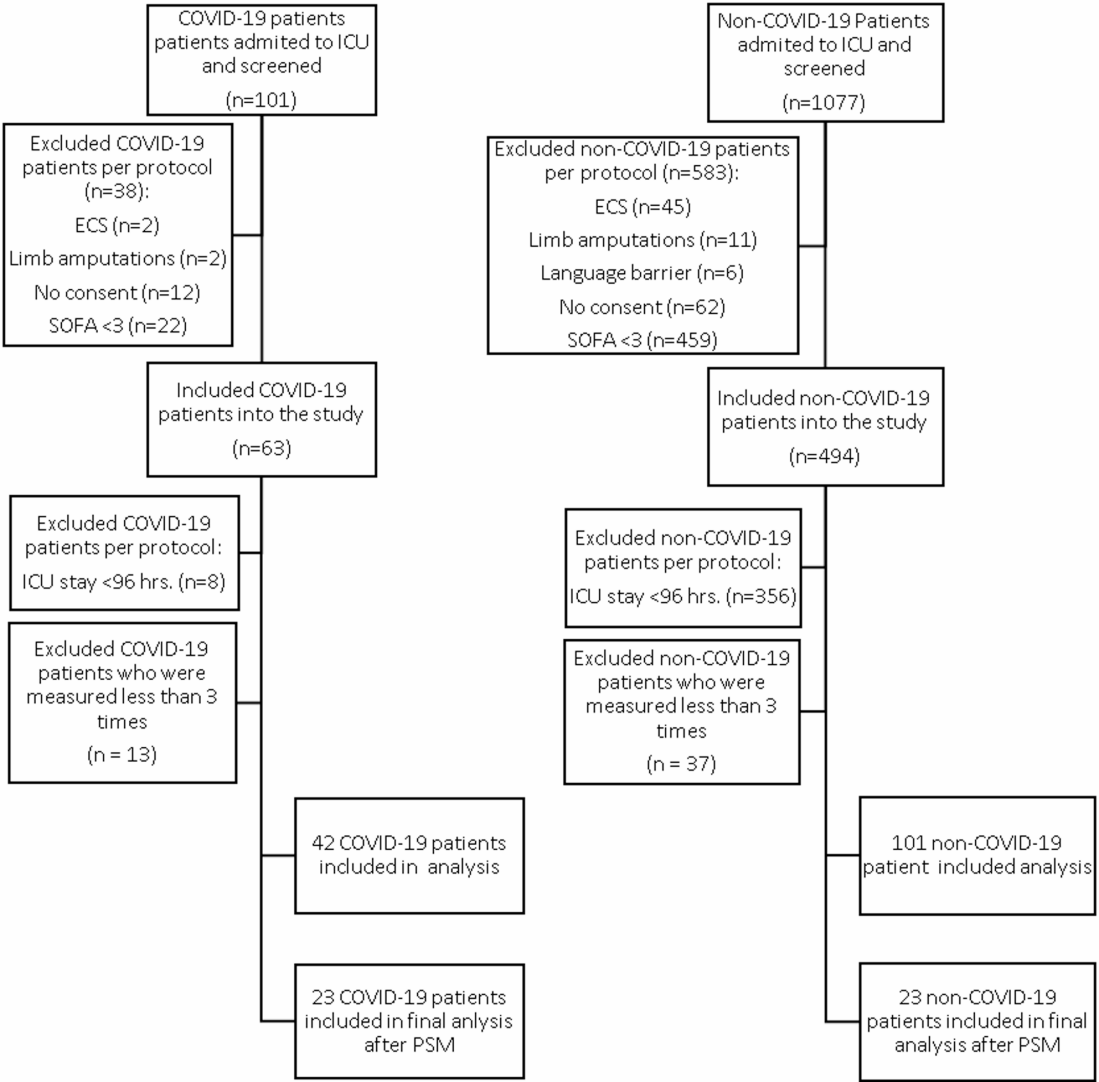
Patient selection flowchart

A total of 1178 patients were screened: 101 were enrolled in the non-COVID-19 group, and 42 were enrolled in the COVID-19 group (Fig. 1). Before matching, substantial baseline differences were observed between groups, including differences in BMI, severity scores, respiratory failure severity and fluid balance (Table 1). Given these substantial baseline differences, inferential analyses focused on the propensity-matched cohort. Detailed descriptive analyses of the unmatched cohort are provided in the Supplementary Material.

**Table 1:** Patient characteristics

Parameter	Unmatched cohort			Matched cohort		
	Patients without COVID-19 (*n* = 101)	Patients with COVID-19 (*n* = 42)	*p* value	Patients without COVID-19 (*n* = 23)	Patients with COVID-19 (*n* = 23)	*p* value
Age, years	55.3 ± 11.31	77.2 ± 8.49	0.333	57.3 ± 10.74	56.6 ± 12.42	0.84
Male sex	73.3%	59.5%	0.115	60.9%	47.8%	0.375
BMI, kg/m^2^	28.9 ± 6.24	33.96 ± 6.92	< 0.0001	27.6 ± 5.78	34.82 ± 8.5	0.002
Admission type						
Medical	57 (56.4)	42 (100)				
Surgical	42 (41.6)	-				
Trauma	2 (2.0)	-				
Admission diagnosis						
Acute pancreatitis	23 (22.8)	-				
Respiratory failure	18 (17.8)	42 (100)				
Post-surgery	17 (16.7)	-				
Liver cirrhosis	14 (13.9)	-				
Sepsis	8 (7.9)	-				
Cardiovascular	7 (6.9)	-				
Metabolic	2 (2.0)	-				
Polytrauma	2 (2.0)	-				
Other	10 (10.0)	-				
APACHE II score	18.2 ± 7.23	12.69 ± 4.64	< 0.0001	16.44 ± 5.26	12.44 ± 4.36	0.007
SOFA score, day 1	7.9 ± 3.17	4.02 ± 2.02	< 0.0001	6.17 ± 3.11	4.44 ± 2.25	0.035
SOFA score, day 7	6.16 ± 3.89	4.36 ± 2.44	0.007	5.0 ± 3.06	4.13 ± 2.6	0.305
NRS-2002 score	3.6 ± 1.30	4.64 ± 1.21	< 0.0001	4.09 ± 1.47	4.65 ± 1.11	0.149
MV	88.0 (87.10)	27 (64.28)	0.003	21 (91.3)	12 (52.2)	0.003
Duration, days	12.1 ± 19.21	9.83 ± 10.07	0.812	11.88 ± 14.83	9.03 ± 9.91	0.449
MV 7 days	73 (72.3)	26 (61.9)	0.238	16 (69.6)	11 (47.8)	0.134
NIV	-	2 (4.76)	-	-	8.7	-
Duration, days		0.83 ± 3.9			0.06 ± 0.21	
HFO _2_	-	36 (85.71)	-	-	82.6	-
Duration, days		4.72 ± 4.2			4.42 ± 4.02	
P/F worst 7 days	114.05 ± 59.02	58.39 ± 15.83	< 0.0001	108.7 ± 49.43	63.22 ± 16.57	< 0.0001
Sedation 7 days	26 (61.9)	77 (76.24)	0.215	17 (73.9) 3.83 (2.93)	12 (52.2) 2.87 (3.22)	0.127 0.298
Use of neuromuscular blocking agents 7 days	35 (34.65)	24 (57.14)	0.001	13 (56.5) 0.96 (1.72)	11 (47.8) 0.96 (1.4)	0.555 1.000
Nutrition						
kcal/kg/d	16.9 ± 5.55	15.89 ± 5.10	0.307	17.6 ± 6.38	16.74 ± 5.64	0.628
Protein g/kg/d	0.8 ± 0.33	0.92 ± 0.37	0.268	0.92 ± 0.35	0.97 ± 0.41	0.609
RRT	42 (41.6)	11 (26.19)	0.083	7 (30.4)	4 (17.4)	0.3
CRP, mg/L	287 ± 157.75	252.47 ± 141.21	0.201	275.33 ± 154.63	233 ± 131.06	0.322
Albumin, g/L	22.8 ± 5.69	25.61 ± 5.07	0.007	21.87 ± 5.69	26.17 ± 4.84	0.009
Total fluid balance on day 5, mL	2330 ± 4420	−616 ± 2749	< 0.0001	2656 ± 3512	−714 ± 2794	0.001
Total fluid balance on day 7, mL	1660 ± 5616	−1181 ± 3500	< 0.0001	2229 ± 3889	−1317 ± 3816	0.003
ICU LOS, days	19.2 ± 19.19	17.12 ± 9.56	0.597	18.04 ± 14.03	16.61 ± 10.05	0.692
Dynamometry, kg	26.83 ± 15.77	24.28 ± 17.44	0.431	24.01 ± 13.6	23.97 ± 15.69	0.431
Left strength %, dynamometry	71.98 ± 36.65	69.09 ± 41.81	0.839	67.65 ± 42.61	71.63 ± 38.62	0.839
ICU mortality	35 (34.70)	15 (35.7)	1.0	9 (39.1)	6 (26.1)	0.345

Data are presented as mean ± standard deviation or n (%)

*BMI* body mass index, *APACHE* Acute Physiology and Chronic Health Evaluation, *SOFA* Sequential Organ Failure Assessment, *NRS-2002* Nutritional Risk Screening 2002, *MV* mechanical ventilation, *NIV* non-invasive ventilation, *HFO*_*2*_ high-flow oxygen therapy, *P/F ratio* PaO_2_/FiO_2_ ratio, *RRT* renal replacement therapy, *CRP* C-reactive protein, *Left strength %*,* dynamometry* calculated residual muscle strength percentage from the norm based on age and sex, *ICU* intensive care unit, *LOS* length of stay, *Matched cohort* after propensity score matching

Propensity score matching (1:1 nearest-neighbour) yielded 23 matched patient pairs. After matching, several important between-group differences persisted. Patients with COVID-19 remained significantly more obese (BMI 34.82 ± 8.50 vs. 27.60 ± 5.78; *p* = 0.002) and continued to have higher illness severity based on APACHE II scores (12.44 ± 4.36 vs. 16.44 ± 5.26; *p* = 0.007) and SOFA day 1 (4.44 ± 2.25 vs. 6.17 ± 3.11; *p* = 0.035). In contrast, SOFA day 7 and NRS-2002 scores no longer differed between groups. Patients with COVID-19 remained more frequently treated with invasive mechanical ventilation (*p* = 0.003), and their P/F ratio continued to be significantly worse (*p* < 0.0001) after matching. In contrast, the use of neuromuscular blocking agents no longer differed between groups (*p* = 0.555). Nutritional intake did not differ between the cohorts (kcal/kg/day or protein g/kg/day). After PSM, albumin concentration remained lower in the non-COVID-19 group (21.87 g/l ± 5.69 vs. 26.17 g/l ± 4.84; *p* = 0.009). COVID-19 patients continued to exhibit a more negative cumulative fluid balance (Day 5: −714 ml vs. + 2656 ml, *p* = 0.001; Day 7: −1317 ml vs. + 2229 ml, *p* = 0.003). Detailed patient characteristics before and after PSM are presented in Table 1.

### Comparison of Pha between groups

**Fig. 2:**
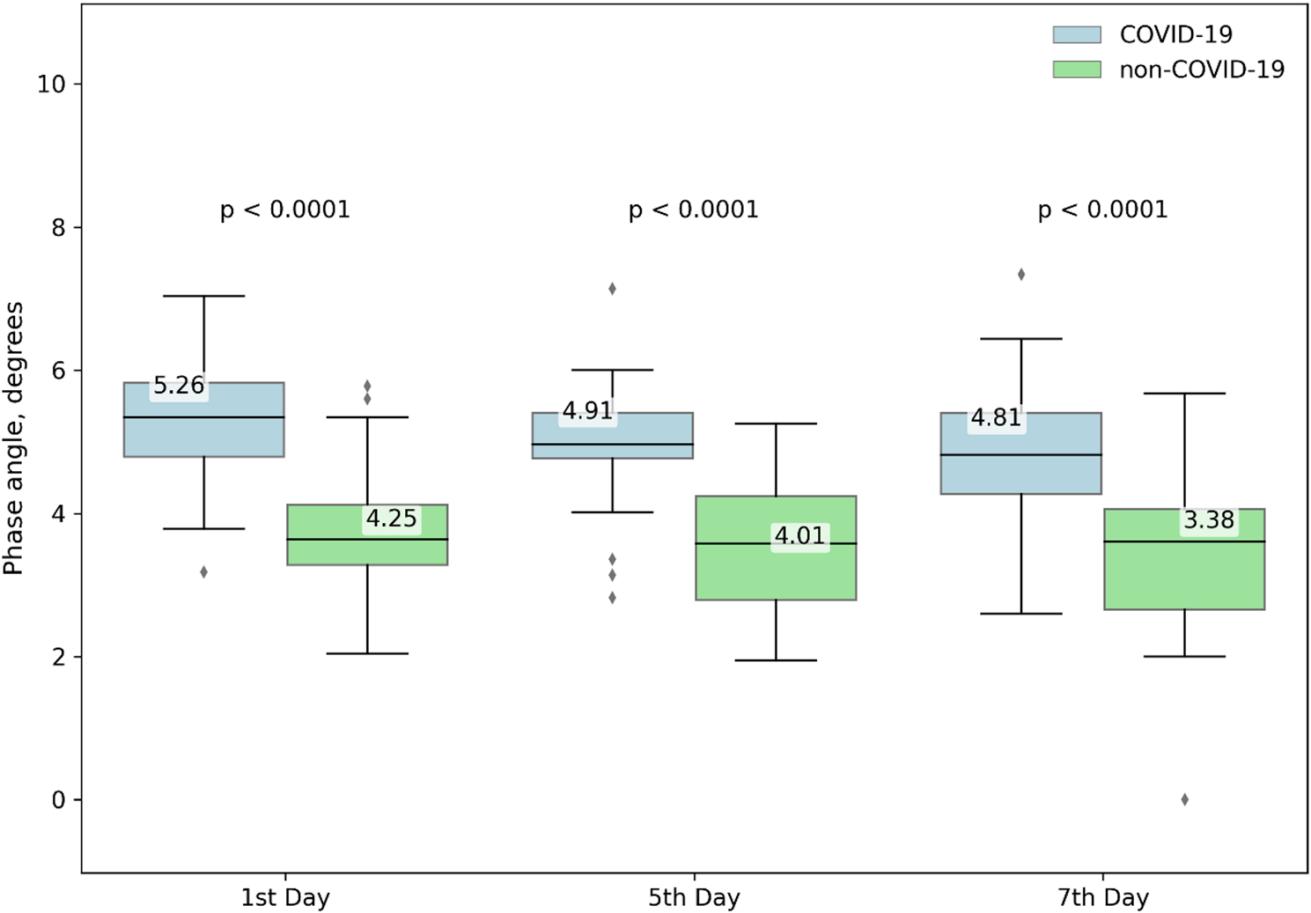
Phase angle differences in matched cohort during ICU stay. Comparison of phase angle on days 1, 5, and 7 of ICU stay in patients with and without COVID-19 propensity score–matched cohort during the first ICU week. Data are presented as box plots. The matched cohort included 23 patients per group (*n* = 23), with all available measurements included in the analysis

After PSM, significant between-group differences in absolute PhA values persisted, with the COVID-19 group consistently showing higher values on day 1 (5.26° ± 0.95 vs. 4.25° ± 1.03; *p* < 0.0001), day 5 (4.91° ± 0.91 vs. 4.01° ± 0.94; *p* < 0.0001), and day 7 (4.81° ± 0.99 vs. 3.38° ± 1.07; *p* < 0.0001) (Fig. 2). However, the percentage change in PhA from day 1 to day 5 (− 6.58% ± 8.15% vs. −6.60% ± 13.46%; *p* = 0.826) and from day 1 to day 7 (− 7.90% ± 17.58% vs. −3.16% ± 17.58%; *p* = 0.473) no longer differed between groups (Supplementary Fig. 4a). After matching, between-group differences in PhA change were no longer statistically significant, and no significant differences were observed in other BIA-derived parameters. Within-group comparisons of PhA between days 5 and 7 again showed no statistically significant change (non-COVID-19: *p* = 0.674; COVID-19: *p* = 0.138).

### Comparison of US measurements between groups

**Fig. 3:**
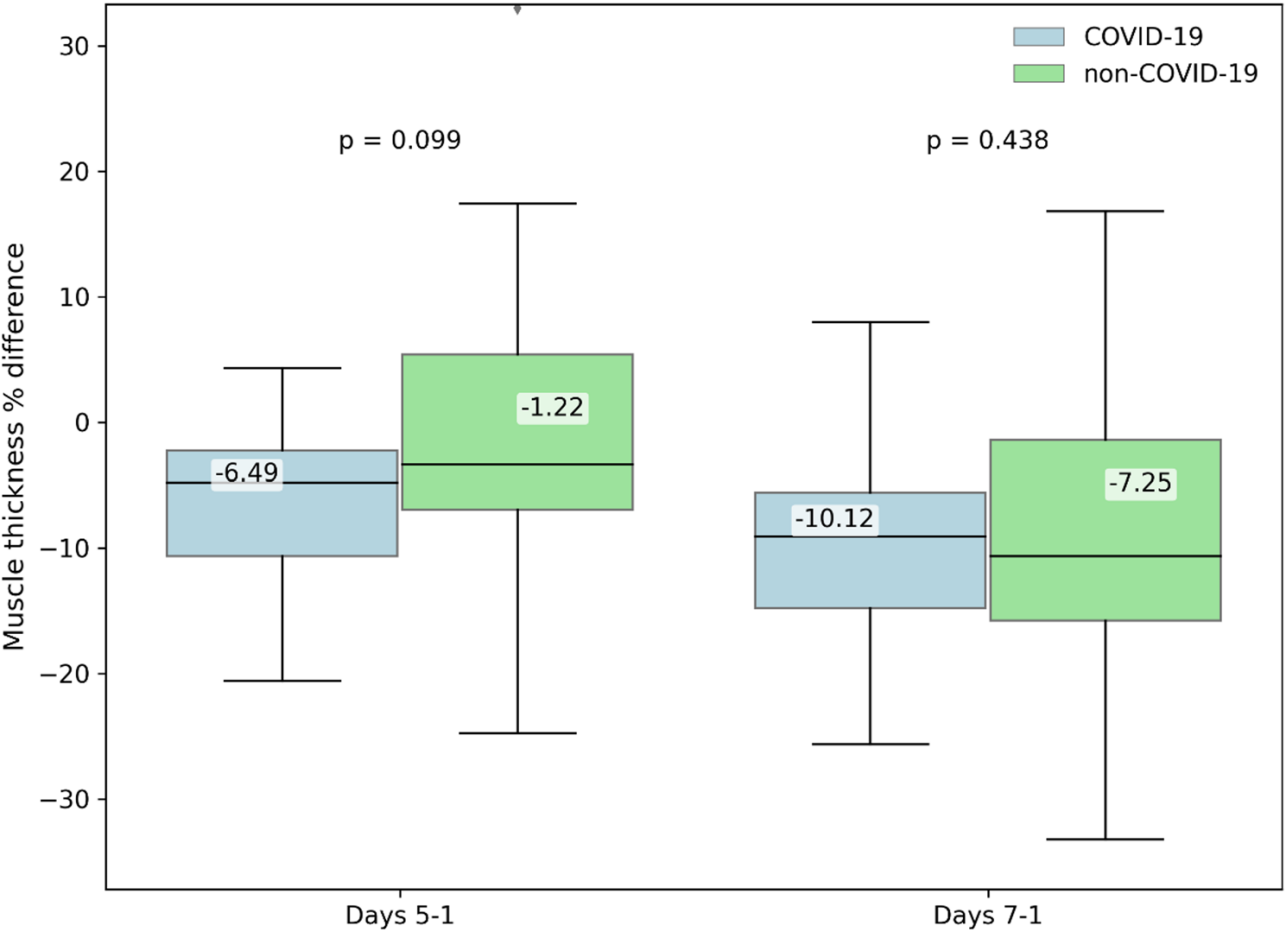
Muscle thickness % differences in matched cohort during ICU stay. Percentage change in ultrasound-derived muscle thickness in the propensity score–matched cohort during the first ICU week. Data are presented as box plots. Measurements were performed on ICU days 1, 5 and 7. Day 1 values were used as baseline The matched cohort included 23 patients per group (*n* = 23), with all available measurements included in the analysis

**Fig. 4:**
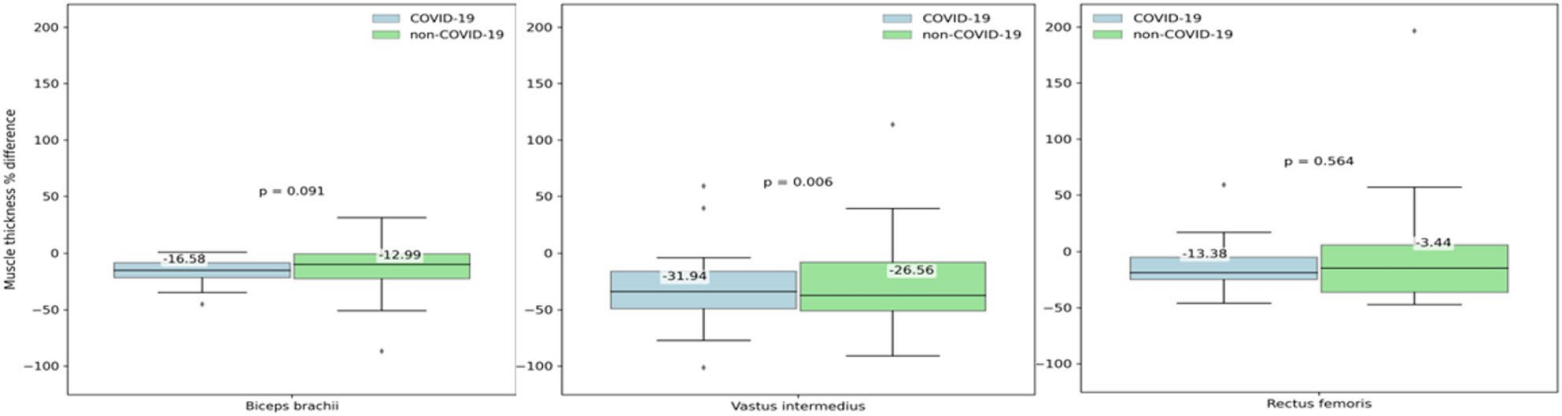
Percentage change in muscle thickness of three individual muscles during ICU stay in the matched cohort. Percentage change in ultrasound-derived muscle thickness of three individual muscles (biceps brachii, rectus femoris and vastus intermedius) in the propensity score–matched cohort during the first ICU week (7 − 1 Day). Data are presented as box plots. Measurements were performed on ICU days 1 and 7. Day 1 values were used as baseline. The matched cohort included 23 patients per group (*n* = 23), with all available measurements included in the analysis

No statistically significant between-group differences were found after PSM in US-derived percentage changes in muscle thickness from day 1 to day 5 (− 6.49% ± 6.58% vs. −1.22% ± 12.77%; *p* = 0.099) or from day 1 to day 7 (− 10.12% ± 8.19% vs. −7.25% ± 11.11%; *p* = 0.438) (Fig. 3). Nonetheless, the pattern of significant within-group muscle wasting persisted in both matched cohorts (non-COVID-19: *p* = 0.003; COVID-19: *p* = 0.018). In the propensity score–matched cohort, muscle-specific analysis showed that statistically significant temporal changes remained only for the vastus intermedius (COVID-19: *p* = 0.043; non-COVID-19: *p* = 0.006), whereas neither the biceps brachii nor the rectus femoris demonstrated significant changes over time (Fig. 4). Unmatched cohort results are shown in Supplementary materials Fig. 4a.

### Comparison of muscle strength measurements between groups

**Fig. 5:**
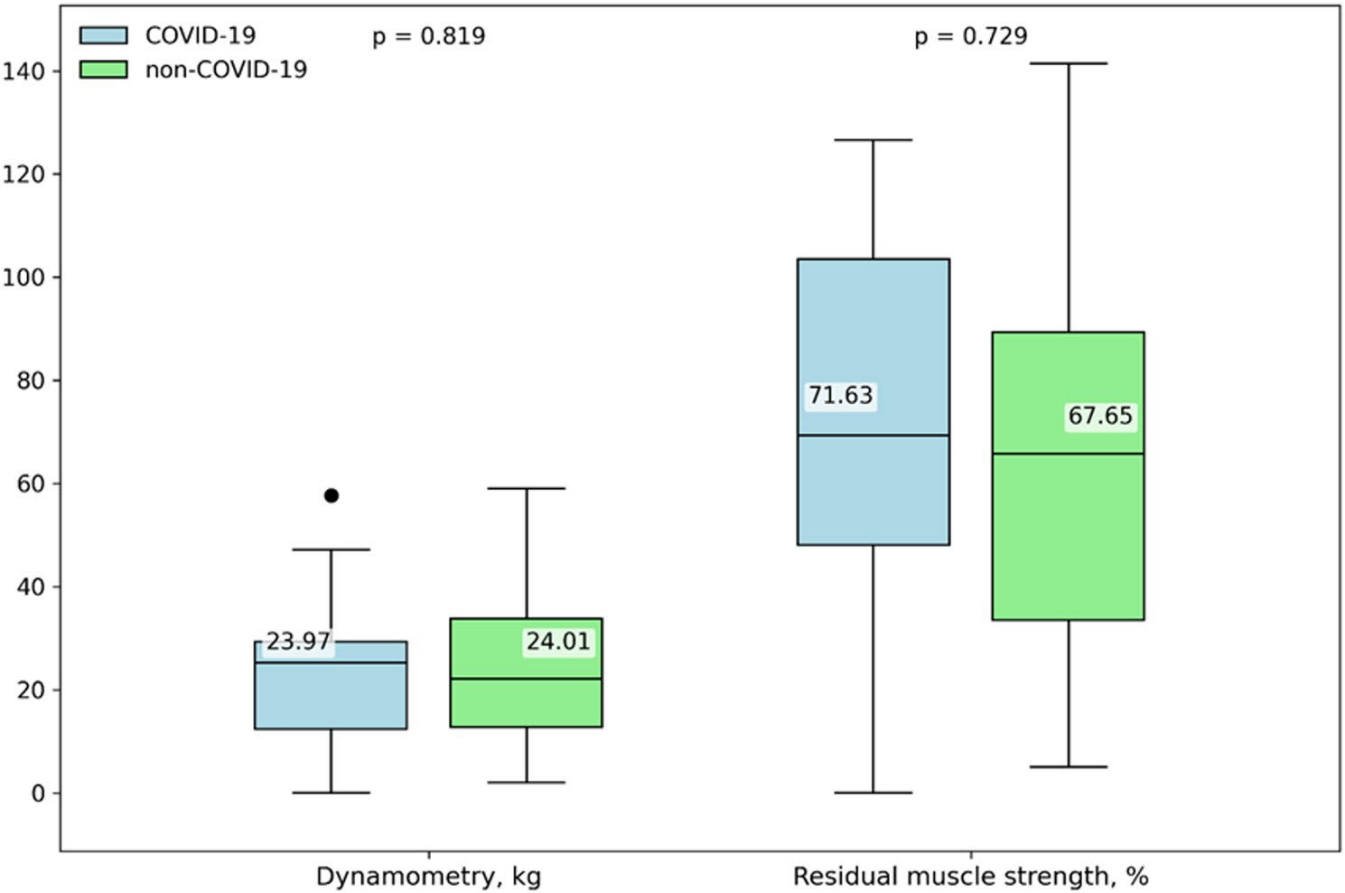
Muscle strength differences measured by dynamometry in matched cohort patients with and without COVID-19. *Dynamometry* muscle strength (kg) in survivors on the day of ICU discharge using a handgrip dynamometer, *Residual percent muscle strength* calculated residual muscle strength percentage from the norm based on age and sex. The matched cohort included 17 patients in COVID-19 and 12 patients in non-COVID-19 group

After propensity score matching, no statistically significant differences were observed between groups. The incidence of muscle weakness remained comparable (non-COVID-19: 27.3% vs. COVID-19: 17.6%; *p* = 0.544). Likewise, neither absolute handgrip strength at ICU discharge (*p* = 0.819) nor residual muscle strength (*p* = 0.729) differed between matched cohorts. Detailed results are shown in Fig. 5.

## Discussion

We conducted a continuation study involving ICU patients with acute respiratory failure due to COVID-19 pneumonia and compared their muscle wasting with that of ICU patients without COVID-19. To minimise baseline confounding and allow for a more accurate comparison of muscle-related outcomes, a PSM analysis was conducted. Although this reduced the sample size, it allowed for a more balanced comparison between the groups. The two cohorts differed significantly. Patients with COVID-19 were more frequently obese—a known risk factor for the disease, as reported by van der Voort et al. [22]—yet had lower severity scores according to the APACHE II and SOFA scales, consistent with findings reported by Wilfong et al [23]. Patients with COVID-19 also required invasive mechanical ventilation less often (64.28% vs. 88.00%) than patients without COVID-19, reflecting the frequent use of high-flow oxygen therapy (HFO₂), which has been shown to reduce intubation rates, hospital-acquired infections, and mortality, as demonstrated in studies by Obradovic et al., Beduneau et al., and Frat et al. [24–26] Nevertheless, owing to poorer P/F ratios, when invasive ventilation was required, patients with COVID-19 more often required deeper sedation and neuromuscular blockade.

Although the duration of mechanical ventilation and ICU LOS did not differ between groups in our study, patients with COVID-19—despite having lower severity scores according to APACHE II and SOFA — they exhibited more severe isolated respiratory failure (as reflected by the P/F ratio), which largely explains their higher need for neuromuscular blockade and negative fluid balance. Whereas the latest meta-analysis by Bellaver et al. did not definitively confirm that neuromuscular blockade increases the risk of ICU-AW, [27] a more recent study by Armestar et al. revealed an association, with an odds ratio of 3.54 [28].

All patients with COVID-19 in our cohort were treated with corticosteroids in line with standard protocols; however, data regarding corticosteroid use in patients without COVID-19 were not collected. According to recent studies, corticosteroids remain a debated risk factor for ICU-AW. Some investigations, including those by Hough et al. and Keh et al., found no definitive association, [29, 30] whereas a recent systematic review by Yang et al. suggested that corticosteroids may contribute to ICU-AW development in certain subgroups of critically ill patients [31]. 

Given the extent of lung injury in patients with COVID-19, a restrictive fluid management strategy was applied, resulting in lower cumulative fluid balances on ICU days 5 and 7. Restrictive fluid therapy is a recommended approach for patients with COVID-19 because it facilitates faster weaning from mechanical ventilation and shortens ICU and hospital stays, as shown in studies by Ahuja et al., Eftekhar et al., and Esper Treml et al. [32–34] In our study, although the COVID-19 group showed a numerically shorter duration of mechanical ventilation, the difference was not statistically significant. Moreover, the ICU LOS and mortality did not differ between groups, an observation that warrants further discussion.

After matching, patients with COVID-19 remained more obese and continued to show higher illness severity (APACHE II and SOFA scores). A statistically significant difference between the groups remained in terms of the P/F ratio and the cumulative fluid balance on days 5 and 7.

Regarding BIA measurements, patients without COVID-19 consistently exhibited worse PhA values across all time points. In a study by Moonen et al. involving 41ICU patients with COVID-19, the admission PhA was 5.2° [35], similar to the value observed in our cohort (5.26°). We also found a statistically significant difference between groups when analysing the percentage change in PhA on days 5 and 7, with the decline being more pronounced in patients with COVID-19. García-Grimaldo et al. reported comparable findings, demonstrating an 8.1%decrease in the PhA over the first 7 days in ICU patients [36], as our study showed a simmilar decline of 7.9%. wo earlier studies in ICU populations without COVID-19 demonstrated that declines in PhA were influenced by increased hydration: Denneman et al. found that a positive cumulative fluid balance by day 7 led to a significant reduction in the PhA (− 0.4°; *p*< 0.01) [37], while another study confirmed similar effects on other BIA-derived parameters [38]. Therefore, BIA results in patients with a positive fluid balance should be interpreted with caution and ideally assessed only after fluid homeostasis has been restored.

In our study, US measurements of muscle thickness did not differ significantly between groups. These findings suggest that US-based assessments of muscle thickness may be less influenced by fluid balance compared with BIA-derived phase angle, particularly in the early phase of critical illness [16]. We observed progressive muscle wasting over time in both groups based on serial US measurements of muscle thickness. Although our methodology differed slightly, Umbrello et al. assessed the rectus femoris cross-sectional area, diaphragmatic thickness, and echogenicity in patients with COVID-19. They reported that non-survivors exhibited a statistically significant reduction in both the rectus femoris cross-sectional area and diaphragmatic thickness during the first 7 days of their ICU stay, together with worsening echogenicity. Their findings suggest that early changes in muscle size and quality may be influenced by nutritional status—particularly cumulative protein deficit—as well as by fluid management strategies [12, 39]. de Andrade-Junior et al. likewise reported an 18.6% reduction in quadriceps muscle thickness, as measured by US, over a 10-day period in patients with severe COVID-19 (*p*< 0.05) [39]. In our cohort, muscle wasting over a 7-day ICU stay reached − 10.12% (*p* = 0.02). However, our patients had lower illness severity as reflected by lower APACHE II and SOFA scores, and the timing of US assessments also differed, which may partially explain the variation in observed muscle loss.

After matching, the between-group difference in PhA dynamics was no longer statistically significant, while changes in muscle thickness measured by US remained non-significant. Within-group analysis showed that changes in the PhA over time were also no longer statistically significant in either group after matching. By contrast, the decrease in muscle thickness assessed by US remained statistically significant over time in both patients with and without COVID-19.

Handgrip dynamometry demonstrated similar absolute muscle strength and residual strength between the groups, consistent with our measurements at ICU discharge showing no between-group differences in absolute strength or residual strength after matching. Although the incidence of muscle weakness was numerically higher in the COVID-19 cohort prior to matching, this difference did not reach statistical significance and became even less pronounced after PSM. Thus, in our study, neither absolute nor residual muscle strength nor the incidence of muscle weakness differed significantly between COVID-19 and non-COVID-19 patients, despite a higher numerical frequency of weakness in the unmatched COVID-19 group. Rahiminezhad et al. reported that ICU-AW was more frequently diagnosed in patients with COVID-19 by day 7, with muscle strength already significantly lower at admission [6]. This may be explained by both direct (via ACE2 receptors and bioactive sphingolipids) and indirect (cytokine storm-mediated) neuromuscular damage, as described by Meacci et al. in their analysis of skeletal muscle involvement in COVID-19. [40] The sensitivity and specificity of handgrip dynamometry for diagnosing ICU-AW exceed 80% when using cut-off values of < 11 kg for men and < 7 kg for women, according to Ali et al. [20] More recent work reaffirmed these findings, although with slightly higher cut-off values of 13.2 kg for men and 9.5 kg for women [19]. Conversely, Piva et al. found no differences between classic acute respiratory distress syndrome and COVID-19–associated acute respiratory distress syndrome survivors, although their study focused on long-term outcomes at 6 and 12 months [41]. In the study by de Andrade-Junior et al., handgrip strength was assessed in a cohort of patients with COVID-19, with longitudinal measurements available for only nine individuals. They observed a significant reduction in handgrip strength (− 22.3%, *p*< 0.05) from days 1 to 10 [39]. In our study, muscle strength and residual strength percentage—adjusted for age- and sex-specific normative values—were evaluated only at the time of ICU discharge. We observed a 30.91% reduction in muscle strength compared with normative values.

Matching did not alter the findings for other BIA-derived, US-based, or handgrip dynamometry measurements, supporting the robustness of these assessment methods across different patient groups.

### Limitations

We must acknowledge several limitations of our study. First, the patient groups differed in disease severity, as reflected by APACHE II and SOFA scores, P/F ratios, frequency of invasive mechanical ventilation, and use of neuromuscular blocking agents. Additionally, the cohort without COVID-19 was heterogeneous, whereas that with COVID-19 consisted exclusively of patients with pneumonia and respiratory failure. The timing of dynamometry measurements also varied among patients because ICU discharge occurred at different times, although the mean ICU LOS did not differ significantly between groups. These factors should be considered when interpreting our findings. Although PSM effectively minimised key baseline differences, it reduced the sample size, which may have limited the ability to detect smaller but clinically meaningful differences between groups. While the matching model included several important confounders—such as age, severity of illness, and exposure to neuromuscular blockade—certain unmeasured factors, including corticosteroid use in patients without COVID-19 and pre-ICU functional status, were not accounted for and may have influenced muscle-related outcomes. As such, the possibility of residual confounding cannot be ruled out. Moreover, the body mass index was deliberately excluded from the matching process because of significant imbalance and limited overlap between groups, which may have constrained our ability to fully explore the impact of obesity on muscle wasting. Further studies involving larger matched cohorts and longer follow-up periods are warranted to better understand the long-term impact of COVID-19–related muscle loss and to inform more individualised rehabilitation strategies for different ICU populations.

## Conclusions

Our study showed that patients both with and without COVID-19 experienced significant muscle wasting and weakness during their ICU stay. Although patients with COVID-19 initially appeared to show more pronounced structural and functional muscle deterioration, these differences were no longer statistically significant after adjustment for baseline severity and treatment differences. This suggests that the development of ICU-acquired muscle weakness is more closely associated with the overall burden of critical illness and intensive care treatment than with the viral infection itself.

In our cohort, ultrasound—particularly measurements of the vastus intermedius—showed consistent temporal declines and therefore appeared more sensitive to detecting early muscle wasting than BIA-derived phase angle, which was probably influenced by hydration and fluid balance. Handgrip dynamometry, assessed at ICU discharge, provided a practical and objective measure of residual muscle function, with comparable strength outcomes between groups after matching.

Future research involving larger, well-matched cohorts and longer follow-up periods is needed to better understand the trajectory of muscle recovery. Such work would support the development of rehabilitation strategies tailored to the specific needs of different ICU populations, including those recovering from COVID-19.

## Supplementary Information

Supplementary Material 1.

Supplementary Material 2.

## Data Availability

The datasets generated and analyzed during the current study are available from the corresponding author on reasonable request **.**.
