# Supplementary material for: Assessment of muscle wasting in intensive care unit patients with and without COVID-19 using ultrasound imaging and bioimpedance analysis
Source: BMC Anesthesiol. 2026 Feb 3;26:156. doi: 10.1186/s12871-026-03659-5 (PMC12958724; doi:10.1186/s12871-026-03659-5)
Supplement: Supplementary file 1 — Supplementary Material 1. [file 12871_2026_3659_MOESM1_ESM.pdf]

## Fig. 2a Phase angle differences in unmatched cohort during ICU stay

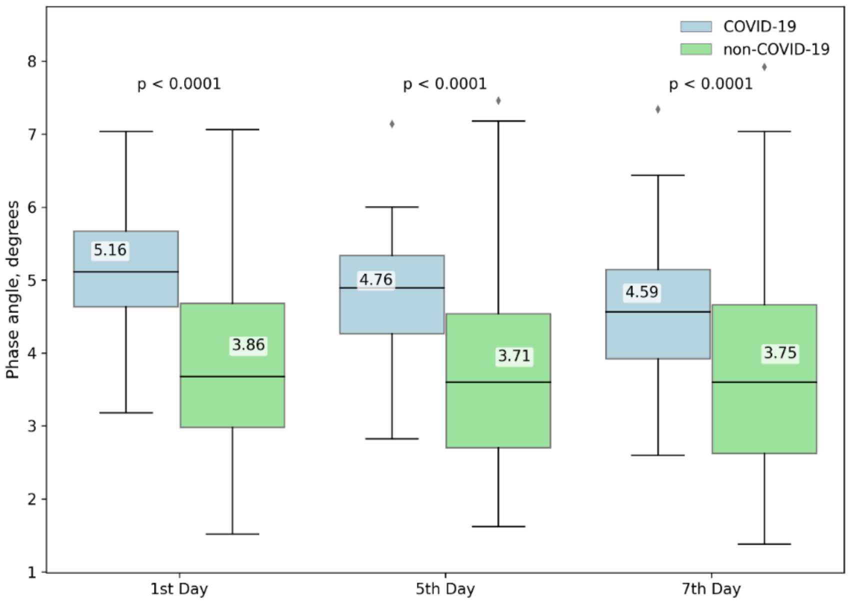


Comparison of phase angle on days 1, 5, and 7 of ICU stay in patients with and without COVID-19 during the first ICU week. Data are presented as box plots. The unmatched cohort included 42 COVID-19 and 101 non-COVID-19 patients, with all available measurements included in the analysis.

## Fig. 3a Phase angle % differences in matched cohort during ICU stay

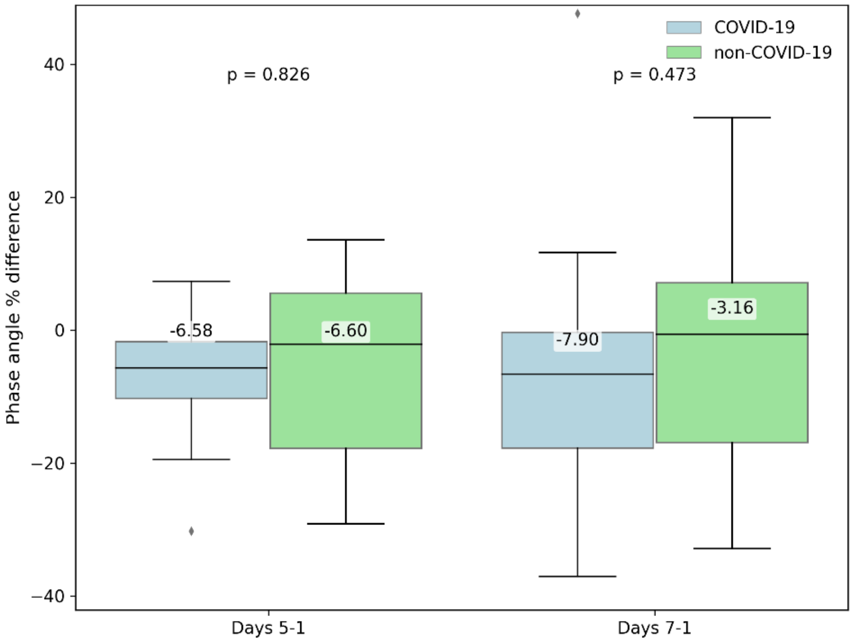

Percentage change in phase angle, measured by BIA in the propensity score–matched cohort during the first ICU week. Data are presented as box plots. Measurements were performed on ICU days 1, 5 and 7. Day 1 values were used as baseline. The matched cohort included 23 patients per group (n = 23), with all available measurements included in the analysis.

## Fig. 3b Phase angle % differences in unmatched cohort during ICU stay

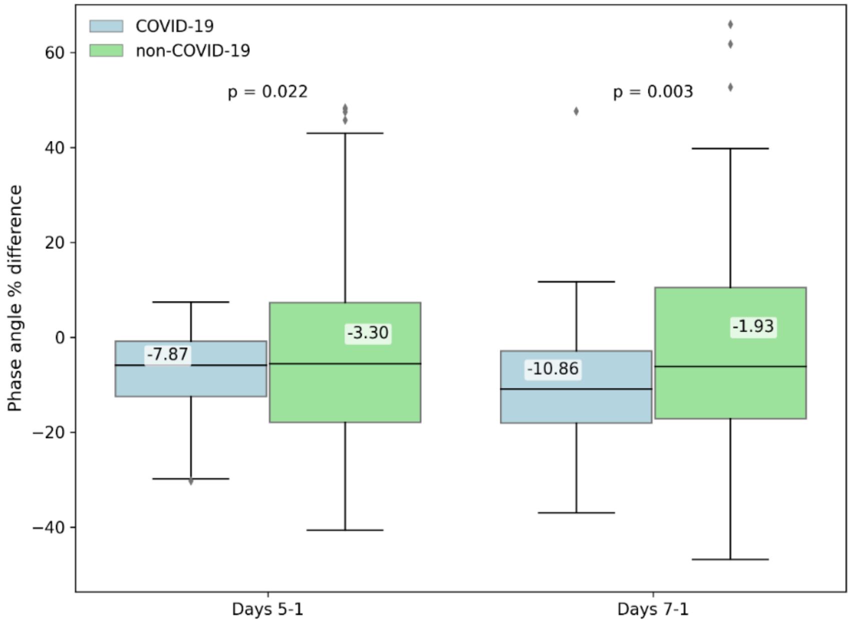


Percentage change in phase angle, measured by BIA during the first ICU week. Data are presented as box plots. Measurements were performed on ICU days 1, 5 and 7. Day 1 values were used as baseline. The unmatched cohort included 42 COVID-19 and 101 non-COVID-19 patients, with all available measurements included in the analysis.

**Fig. 4a Muscle thickness % differences in unmatched cohort during ICU stay**

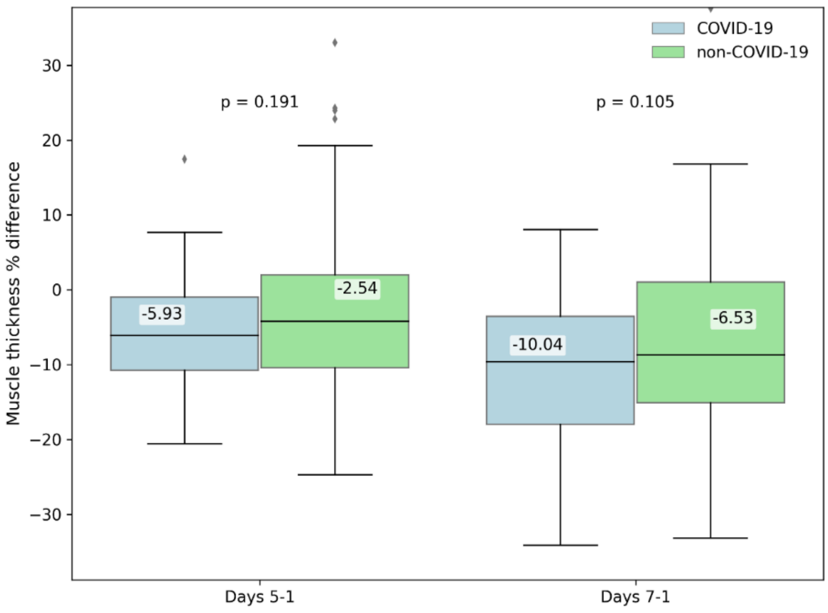


Percentage change in ultrasound-derived muscle thickness during the first ICU week. Data are presented as box plots. Measurements were performed on ICU days 1, 5 and 7. Day 1 values were used as baseline. The unmatched cohort included 42 COVID-19 and 101 non-COVID-19 patients, with all available measurements included in the analysis.

**Figure 5a. Percentage change in muscle thickness of three individual muscles during ICU stay in the unmatched cohort**

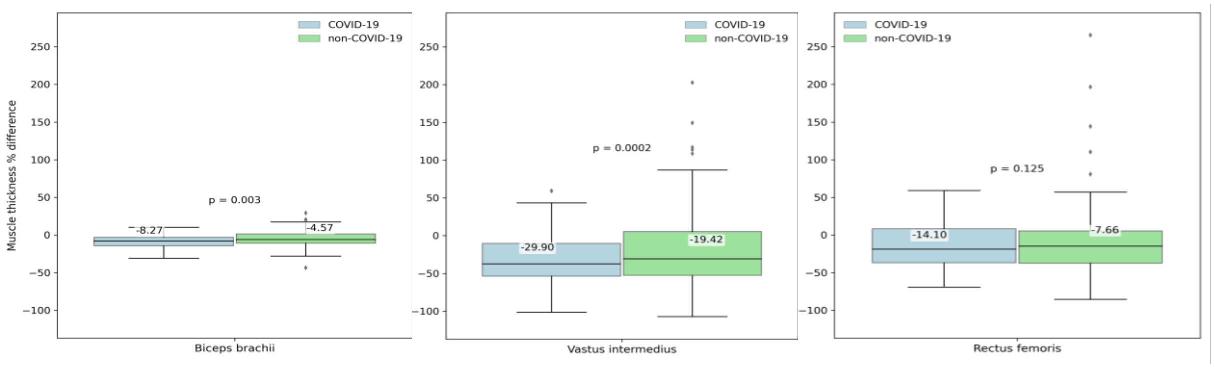


Percentage change in ultrasound-derived muscle thickness of three individual muscles (biceps brachii, rectus femoris and vastus intermedius) during the first ICU week (7-1 Day). Data are presented as box plots. Measurements were performed on ICU days 1 and 7. Day 1 values were used as baseline. The unmatched cohort included 42 COVID-19 and 101 non-COVID-19 patients, with all available measurements included in the analysis.

**Fig. 6a Muscle strength differences measured by dynamometry in unmatched cohort patients with and without COVID-19**

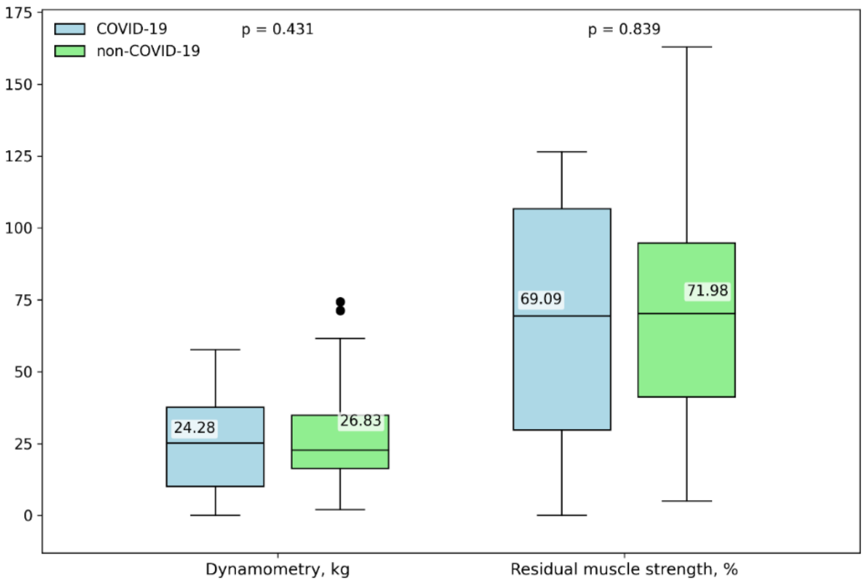


*Dynamometry* muscle strength (kg) in survivors on the day of ICU discharge using a handgrip dynamometer, *Residual percent muscle strength* calculated residual muscle strength percentage from the norm based on age and sex. The unmatched cohort included 27 patients in COVID-19 and 61 patients in non-COVID-19 group. The incidence of muscle weakness at ICU discharge, defined by handgrip dynamometry, was numerically higher in the unmatched COVID-19 group compared with the non-COVID-19 (25.9% vs. 9.8%), although this difference did not reach statistical significance (p = 0.099).
